# 

**Published:** 1894-01

**Authors:** 


VOL. XV.] *PUBLISHED MONTHLY AT THREE DOLLARS A YEAR.* *SINGLE COPIES, THIRTY CENTS.* [NO. 1.

Entered at the Post Office, February 3rd, 1891, at New York, as Second-class Matter.
Copyright, 1893, by W. A. Conklin and R. S. Huidekoper.
Printed for the Editors by E. SCOTT Co., 134 West 23d Street, New York.

### THE JOURNAL

OF

### COMPARATIVE MEDICINE

AND

### VETERINARY ARCHIVES.

EDITED BY

W. A. CONKLIN, Ph. D., D. V. S.

RUSH SHIPPEN HUIDEKOPER, M. D., Veterinarian, (Alfort,)

*NEW YORK.*

JANUARY, 1894.

All communications and payments should be addressed to

**MANAGING EDITOR JOURNAL COMP. MEDICINE AND VET. ARCH.**

Station G, New York City.

Copies may be had in London of BALLIERE, TINDALL & COX.

